# PON-SC – program for identifying steric clashes caused by amino acid substitutions

**DOI:** 10.1186/s12859-017-1947-7

**Published:** 2017-11-29

**Authors:** Jelena Čalyševa, Mauno Vihinen

**Affiliations:** 1 Protein Structure and Bioinformatics, Department of Experimental Medical Science, Lund University, BMC B13, SE-22 184 Lund, Sweden; 2 Present address: EMBL Heidelberg, Meyerhofstraße 1, 69117 Heidelberg, Germany

**Keywords:** Amino acid substitution, Variation interpretation, Structural clashes, Side chain rotamers

## Abstract

**Background:**

Amino acid substitutions due to DNA nucleotide replacements are frequently disease-causing because of affecting functionally important sites. If the substituting amino acid does not fit into the protein, it causes structural alterations that are often harmful. Clashes of amino acids cause local or global structural changes. Testing structural compatibility of variations has been difficult due to the lack of a dedicated method that could handle vast amounts of variation data produced by next generation sequencing technologies.

**Results:**

We developed a method, PON-SC, for detecting protein structural clashes due to amino acid substitutions. The method utilizes side chain rotamer library and tests whether any of the common rotamers can be fitted into the protein structure. The tool was tested both with variants that cause and do not cause clashes and found to have accuracy of 0.71 over five test datasets.

**Conclusions:**

We developed a fast method for residue side chain clash detection. The method provides in addition to the prediction also visualization of the variant in three dimensional structure.

**Electronic supplementary material:**

The online version of this article (10.1186/s12859-017-1947-7) contains supplementary material, which is available to authorized users.

## Background

Amino acid substitutions (AASs) are common variants and can have numerous effects and mechanisms [1]. A large number of prediction methods is available for investigating the tolerance of variants [2–4] as well as their mechanisms including effects on protein stability [5–7], disorder [8], aggregation [9, 10], localization [11], interactions, electrostatics, RNA splicing [12, 13], tRNA molecules [14, 15] etc. [16, 17]. Specific predictors are available for variants in some proteins including BRCA1 and 2 [18, 19], mismatch repair system proteins [20, 21], and Bruton tyrosine kinase (BTK) [22]. Recently it has become possible to predict also the phenotypic severity of disease-related variants [23].

Among the most common effects are structural alterations originating because the substituted residue cannot fit into the structure without causing (major) structural alterations. When the substituting residue does not fit in the structure, more or less drastic conformation change occurs as the consequence. Due to structural and physical reasons all side chain conformations are not possible or structurally favorable, instead there are certain most favored conformations called for rotamers. Structural alterations may occur due to several other reasons including new or deleted interactions such as salt bridges or disulfide bonds, altered ligand binding specificity and modified allosteric site.

Libraries of side chain rotamers have been determined either from crystal structures [24, 25] or based on molecular dynamics simulations [26]. These libraries contain residue rotamers independent of the backbone conformation or dependent on the local backbone, especially secondary structures. Methods have been described for side chain optimization [27, 28]. These tools typically utilize a rotamer library, then apply an energy function to estimate rotamers and search algorithm to cover the three dimensional space.

Only a few tools have been developed for the prediction of the effect of AASs on protein structure [29–31]. These methods are either not available, do not have easy to use interface, or they are too slow to apply to large datasets, such as those generated by modern next generation sequencing (NGS) techniques. Methods for optimizing the side chain rotamers could be used for the task; however they are not designed to answer this question. To fill the gap, we developed a novel and fast method, PON-SC, to predict whether AASs are structurally compatible or if they form clashes. The method is applicable both for protein engineering applications when planning either stability increasing [32–34] or decreasing [35, 36] variations, as well as for the interpretation of variants [22, 37, 38]. If the introduced variant cannot be accommodated into the structure without severe clashes and consequent structural alterations, the variant is harmful, even disease-causing. The performance of the method was benchmarked with known harmful and structurally compatible cases that were collected from several sources.

## Method

**Fig. 1:**
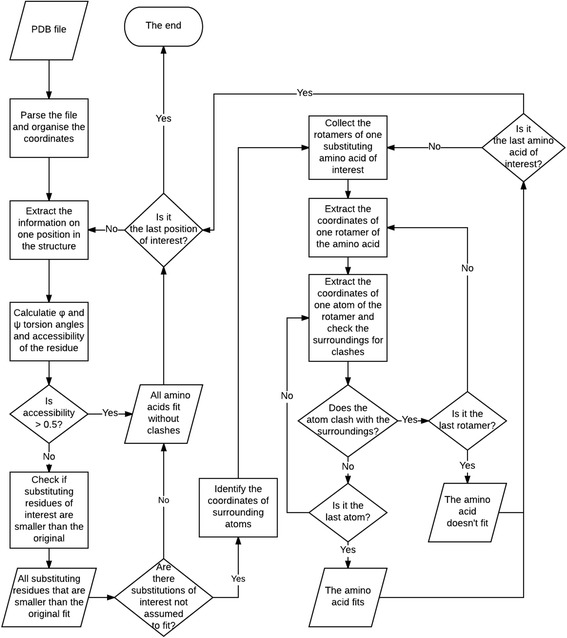
The scheme of the method to identify amino acid substitutions causing clashes. Using PDB file as an input, the program iterates through all positions of interest in the structure, making assumptions and performing calculations for every substitution of interest, and providing information on whether the amino acid substitutions cause clashes in the structure or not

A novel method was developed for side chain clash detection. The flowchart of the protocol is shown in Fig. 1. PON-SC analysis is based on fitting AASs to protein structures, thus three dimensional structure is needed. Even structural models can be used, but then it is up to the user to estimate how reliable the predictions are.

The method has decision points depending on the submission and prediction request (Fig. 1). The predictor was programmed with Python. Two approaches are used to make decisions about side chain compatibility; assumptions based on the location and type of the original and substituting residue as well as rotamer testing predictions.

### Processing of the input

BioPython package [39] is used to parse the input file in PDB format. φ and ψ torsion angles of amino acid backbones and accessibility of the side chains are calculated with STRIDE [40]. KDTree algorithm from scikit-learn package [41] is used to prepare the structures for rotamer tests. The amino acid side chain rotamers are added to the C_α_ atom of the substituted residue.

### Assumptions about side chain alterations

We use backbone-dependent rotamer library [24] for testing the space of potential side chain conformations. For φ and ψ torsion angles of the selected residue, common rotamers for the substituting amino acid are considered.

To simplify and speed up the calculations, the following assumptions are made. First, if the ratio between the accessibility of the original residue and the highest possible accessibility of that residue type [42] is ≥0.5 and the side chain is 3 or more heavy atoms long, all substitutions are assumed to fit into the structure. Thus, the method finds accessible positions that structurally allow all changes. Second, when the original amino acid is larger than the substituting one, no clashes are expected. Glycine is allowed in all positions, and smaller than original residues throughout the structure if they have a fitting structure. As an example, valine or leucine which have short but branched side chains are not directly assumed to be able to replace e.g. for arginine or lysine which have longer side chains. In these cases, the method tests whether the amino acid rotamers fit into the structure.

### Identifying fitting amino acids with calculations

**Fig. 2:**
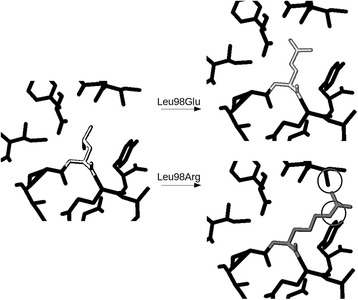
An example of a clash between atoms caused by amino acid a substitution. Substitution of Leu98 (white) by Glu (top) in SH2D1A protein (PDB id 1D4W) causes no clashes with the surrounding residues, while substitution with Arg (bottom) causes clashes with Ile84 and Tyr29 (indicated by circles)

The furthermost possible clash is calculated to be in the distance to the N_ƞ_ atom of the straightest possible conformation of arginine and adding the van den Waals radius of nitrogen (1.64 Å). Hydrogen atoms are ignored in the calculations. Variants left after the initial test are fitted in the available space around the residue. Side chain rotamers are tested to find one that fits into the structure. If the residue does not have any rotamer that would fit the substitution, it is considered to cause a clash and to be harmful (Fig. 2).

To calculate if a rotamer fits in the available space, rotation matrix for that rotamer is calculated and the clash detection is initiated. All atoms in the surroundings that possibly could form a clash are considered. For every atom starting after C_β_ (atom1), the clash with every atom in the surrounding space (atom2) is calculated as follows:

$$c={r}_{atom1}+{r}_{atom2}-{d}_{atom s}-{d}_{allowed},$$

where *c* is the size of the overlap between the atoms, *r*_*atom*_ is the van der Waals radius of the atom, *d*_*atoms*_ is the distance between two atoms, and *d*_*allowed*_ is the allowed clashing distance. The default *d*_*allowed*_ value is 0.4 Å [42]. The sum of the radii of atoms is set to 2.5 Å when they form a hydrogen bond [43]. If both atoms are a part of cysteine side chain, the calculation is adjusted so that the minimal allowed distance between C_α_ atoms is 4 Å [44]. If the clash value is positive, the rotamer is discarded as not fitting and a new rotamer is taken until all of them have been tested or a fitting one is found.

### Datasets for validating the method

The method was tested with structures from the PDB database [45]. First, PDB structure pairs differing by one amino acid were identified. After cleaning the data from incompatible PDB entries that either lacked information or when the positions in the structures did not match with the positions in corresponding protein sequences, the final set of 7795 variations was obtained. All the datasets used in this paper are available at VariBench database for variation prediction and testing database [46]. For comparison, clashing substitutions were identified by coupling SCWRL4 [27] and Probe [47] programs. SCWRL4 was used to build structures with the variant residues and Probe to detect clashes in them.

To further validate the method, several known cases of AASs having clashes were used. These included variants in CD40 ligand that is expressed in lymphocytes [37]. The structural effects of AASs were studied by bioinformatics methods in the structure of CD40LG tissue necrosis factor (TNF) homology domain (PDB ID 1ALY). 13 variations were reported to cause conformational damage and 19 not to affect the structure negatively.

Another dataset was for pathogenic Src homology 2 (SH2) domain variations in 12 SH2 domains in 8 proteins [48]. The structures included the SH2 domain-containing 1A (SH2D1A), the zeta chain of T cell receptor associated protein kinase 70 (ZAP70) N-terminal SH2 domain, the phosphoinositide-3-kinase regulatory subunit 1 (PIK3R1) SH2 domain, the signal transducer and activator of transcription 1α (STAT1) SH2 domain, the BTK SH2 domain, and the RAS p21 protein activator 1 (RASA1) SH2 domain with corresponding PDB IDs 1D4W, 1M61, 2IUG, 1YVL, 2GE9 and 2GSB, respectively. Totally 28 structurally incompatible and 71 structurally compatible or neutral variations were obtained.

For human elastase, neutrophil expressed (ELANE, 1PPF) 23 AASs of which 3 were structurally compatible were obtained [38]. Variants in tumor protein p53 (TP53) [29] were included. There are 94 structures in the PDB database for the TP53 core domain/DNA complex, staphylococcal nuclease and the SH3 domain, PDB IDs 1TSR, 1STG and 1FMK, respectively. Totally 43 AASs cause clashes, while the number of amino acid substitutions not causing clashes is 121.

Colorectal and breast cancer variations in TP53, KRAS proto-oncogene, GTPase (KRAS) and SMAD family member 4 (SMAD4) (1TSR, 1DD1 and 3GFT) [49] have been investigated at structural level. 10 out of the 31 studied substitutions were found to cause steric clashes.

All the datasets are available at VariBench at http://structure.bmc.lu.se/VariBench/sidechain.php.

### Performance measures

The method performance was assessed by using six performance scores [50] following guidelines for reporting [51]. When TP is the number of clash-causing variations predicted as not fitting into the structure, TN is the number of structure compatible variants that fit into the structure, FP is the number of fitting variations predicted as causing clash and FN is the number of clashing variations predicted as fitting into structure, the equations for computing the six performance measures are as follows:

$$Accuracy=\frac{\ \mathrm{TP}+\mathrm{TN}}{\mathrm{TP}+\mathrm{TN}+\mathrm{FP}+\mathrm{FN}},$$

Positive predictive value.

$$PPV=\frac{\ \mathrm{TP}}{\mathrm{TP}+\mathrm{FP}},$$

Negative predictive value

$$NPV=\frac{\ \mathrm{TN}}{\mathrm{TN}+\mathrm{FN}}$$

Sensitivity/True positive rate

$$TPR=\frac{\ \mathrm{TP}}{\mathrm{TP}+\mathrm{FN}}$$

Specificity/True negative rate

$$TNR=\frac{\ \mathrm{TN}}{\mathrm{TN}+\mathrm{FP}}$$

and Matthews Correlation Coefficient

$$MCC=\frac{\ \left(\mathrm{TP}\times \mathrm{TN}\right)\hbox{-} \left(\mathrm{FP}\times \mathrm{FN}\right)}{\sqrt{\left(\mathrm{TP}+\mathrm{FN}\right)\times \left(\mathrm{TP}+\mathrm{FP}\right)\times \left(\mathrm{TN}+\mathrm{FN}\right)\times \left(\mathrm{TN}+\mathrm{FP}\right)}},$$

where NPV is negative predictive value and PPV is positive predictive value and MCC is Matthews correlation coefficient.

## Implementation

The program has web interface that was programmed with Python using Django platform. There are several options for submitting variants. By providing PDB ID, the structure will be downloaded from PDB. Users need to note that PON-SC will consider clashes with all atoms in the PDB file. It may be necessary to exclude solvent atoms other than waters, which are automatically excluded from the calculations. It is possible to submit variants in several proteins at one time. Further, the user can choose to submit own PDB coordinates.

The variants to be analyzed are listed one per line. If only the position number is provided PON-SC predicts all 19 AASs in that position. The variant visualizations are available by JavaScript Protein Viewer (https://biasmv.github.io/pv/). The results can be obtained while waiting or by e-mail. PON-SC is freely accessible at http://structure.bmc.lu.se/PON-SC.

## Results and discussion

To identify AASs causing clashes in structures, various properties of the amino acids and polypeptides have to be considered. These include different radii of interacting atoms, bond lengths, hydrogen and disulfide bonds, the limited flexibility of the side chain in the structure, errors in resolved protein structures, etc. PON-SC considers clashes if the substituting residue comes too close to other atoms in the structure. The method considers clashes also with ligands and heteroatoms, if included to the structure. Waters are automatically removed from the calculations.

### Performance of the program

PON-SC is very fast, it takes on average 0.05 s to evaluate a substitution once the PDB file is downloaded. SCWRL4 [27] is a widely used method for side chain rotamer optimization. It is used together with Probe [47], an atomic packing evaluation tool, to detect clashes. These programs are substantially slower than PON-SC because several intermediate steps are required e.g. to create new protein structures for every amino acid substitution and parsing the outputs of the programs. Calculation for a variant takes on average 1.3 s per substitution for SCWRL4 + Probe, i.e. it is 26 times slower than PON-SC. Note that SCWRL4 and Probe are not combined into a package, instead are run separately. SCWRL4 and PON-SC use the same rotamer library.

We tested the method performance with datasets of known cases. Data for clashes are limited as there are usually no structures for them. AASs that are clash-free were collected by identifying PDB structures that had only one residue difference. 7795 such cases were found and predicted both with PON-SC and SCWRL4 + Probe (Table 1). 77.4% of these AAS were predicted by PON-SC not to cause clashes. The performance of SCWRL4 + Probe is somewhat higher, having correct predictions in 83.6% of the cases. This test was made to address how many negative cases i.e. tolerated AASs are correctly predicted.

**Table 1:** Number of predicted clashes by amino acid types in PDB structures that tolerate substitutions

	PON-SC number	PON-SC (%)	SCWRL+ Probe number	SCWRL+ Probe (%)	Both^a^ number	Both (%)	Total^b^
Alanine	0	0	0	0	0	0	1165
Arginine	86	25.52	11	3.26	15	4.45	337
Asparagine	37	8.22	112	24.89	7	1.56	450
Aspartic acid	38	8.35	125	27.47	13	2.85	455
Cysteine	0	0	4	1.316	0	0	304
Glutamic acid	92	18.70	42	8.54	42	8.54	492
Glutamine	42	15	31	11.07	22	7.86	280
Glycine	0	0	0	0	0	0	393
Histidine	74	21.70	45	13.20	38	11.14	341
Isoleucine	94	33.45	38	13.52	42	14.95	281
Leucine	96	25.26	39	10.26	27	7.11	380
Lysine	53	19.41	6	2.20	5	1.83	273
Methionine	93	32.63	11	3.86	10	3.51	285
Phenylalanine	86	18.86	118	25.88	95	20.83	456
Proline	90	76.92	0	0	0	0	117
Serine	5	0.89	4	0.71	0	0	561
Threonine	78	24.68	30	9.49	28	8.86	316
Tryptophan	54	29.19	29	15.68	58	31.35	185
Tyrosine	110	30.05	76	20.77	71	19.40	366
Valine	122	34.08	41	11.45	42	11.73	358

^a^Does not include cases listed in PON-SC and SCWRL+Probe columns. ^b^Total number of substitutions in the dataset.

The reason for detecting clashes among these cases is at least partly due to structural rearrangements outside the variant position. Alterations due to AASs can appear in several amino acids [29, 52] not only in the substitution site. Neither PON-SC nor SCWRL + Probe combination can detect these. However, SCWRL4 + Probe performs better since SCWRL4 allows flexibility for the backbone and side chain as it is an optimization tool.

### Performance for different AASs

Neither of the methods had problems fitting smaller amino acids in the available space in the structure (Table 1). Substitutions to alanine or glycine did not cause clashes. Substitutions to cysteine and serine formed clashes only in a few cases. The reason behind SCWRL4 + Probe identifying clashes in the case of introducing cysteine could be that the method didn’t account for disulfide bridges in the structure. PON-SC did not have any problems with substitutions to cysteine.

In the case of substitutions to larger amino acids, the situation is more variable. Some of the differences between the methods can be explained by the higher flexibility allowed by SCWRL4 including alterations to the polypeptide backbone. Proline is the most problematic side chain for PON-SC to predict. This is because the method provides freedom only for side chains, whereas in proline substitutions also the backbone is altered. Therefore, the method over-predicts clashes in proline substitutions.

In case of asparagine, aspartic acid and phenylalanine PON-SC identified far less clashes than SCWRL + Probe. Interestingly, the situation is the opposite for the related substitutions by glutamine and glutamate. In conclusion, the two approaches, PON-SC and SCWRL4 + Probe, performed overall quite similarly; however, there were major substitution type-specific differences.

### Comparison to previous studies of steric clashes

A real test for a predictor is to use both positive and negative cases. We collected five datasets from different studies. Since protein structures with major clashes cannot be investigated with e.g. crystallography and since negative results are not frequently published, there are not many cases with reported clashes in literature and databases. After extensive search we found five datasets that we used to test the performance of the tool.

The average performance over all the datasets is as follows: sensitivity is 0.66, specificity 0.77, accuracy 0.71 and MCC 0.43 (Table 2). Only the datasets for TP53 and cancers have specifically addressed the clashes of the substitutions. PON-SC has typically higher specificity than sensitivity, i.e. it predicts clashes with somewhat lower accuracy than tolerated variants. Exception is the ELANE dataset, but since this is a small set, minor random effects may have major impact. The average accuracy of 0.71 indicates that the method is rather reliable, and because of its speed, it can thus be used for analysis of even large datasets. The overall quality scores are more relevant since the individual datasets are quite small.

**Table 2:** Validation of the method performance

Study	TP	FP	TN	FN	Total	NPV	PPV	Sensitivity	Specificity	Accuracy	MCC
CD40LG	9	1	18	4	32	0.75	093	0.69	0.95	0.82	0.66
SH2	13	25	46	15	99	0.54	0.57	0.46	0.65	0.55	0.11
ELANE	16	1	2	4	23	0.77	0.70	0.80	0.65	0.73	0.46
TP53	27	19	102	16	164	0.69	0.80	0.63	0.84	0.74	0.48
CANCER	7	5	16	3	31	0.72	0.75	0.71	0.76	0.74	0.47
Total/Average	72	51	184	42	349	0.69	0.74	0.66	0.77	0.71	0.43

The PON-SC program does not give information on the severity of a clash, only that it occurs. The method is implemented such that the detected clashes are highly likely structurally incompatible and therefore harmful. For visualization of the results the PON-SC tool utilizes the JavaScript Protein Viewer plugin that shows the original and variant residues in three dimensional structures. The rotamer used for the visualization is not necessarily the best fitting one but it is the most common of the fitting ones, as the rotamers are tested in the decreasing order of frequency. To save time, the program ends the search after finding the first fitting rotamer and then that one is visualized. For the prediction purposes it is sufficient to find one rotamer that allows fitting the novel side chain.

For comparison, the results for the SCWRL + Probe are shown in Additional file 1: Table S1. On these datasets PON-SC has somewhat better performance and also displays more balanced results in regards to the measures. The MCC and accuracy are 0.29 and 0.43, and 0.64 and 0.71 for SCWRL + Probe and PON-SC, respectively. PON-SC had equal or better values for all the five tested variation sets.

## Conclusions

PON-SC is a novel method for varient effect prediction. It detects structural clashes due to AASs based on protein three dimensional strucutre, side chain rotamer library, structural assumptions and calculations. The method has a relatively high performance, accuracy being 0.71 over several datasets. PON-SC is currently the only tool that can be used for large scale analysis e.g. of NGS datasets. Side chain replacements can be visualized in protein structures.

## Availability and requirements

Project name: PON-SC.

Project home page: http://structure.bmc.lu.se/PON-SC

Operating system(s): Linux.

Programming language: Python.

Any restrictions to use by non-academics: contact authors.

## Additional file

Additional file 1: Table S1. Results for SCWRL + PROBE on validation dataset. (PDF 13 kb)

## Abbreviations

AAS: Amino acid substitution
BTK: Bruton tyrosine kinase
ELANE: Elastase, neutrophil expressed
FN: False negative
FP: False positive
KRAS: KRAS proto-oncogene, GTPase
MCC: Matthews correlation coefficient
NGS: Next generation sequencing
NPV: Negative predictive value
PIK3R1: Phosphoinositide-3-kinase regulatory subunit 1
PPV: Positive predictive value
RASA1: RAS p21 protein activator 1
SH2D1A: SH2 domain-containing 1A
SMAD4: SMAD family member 4
STAT1: Signal transducer and activator of transcription 1
TN: True negative
TNF: Tissue necrosis factor
TNR: True negative rate
TP: True positive
TP53: Tumor protein p53
TPR: True positive rate
ZAP70: Zeta chain of T cell receptor associated protein kinase 70
