# Supplementary material for: PON-SC – program for identifying steric clashes caused by amino acid substitutions
Source: BMC Bioinformatics. 2017 Nov 29;18:531. doi: 10.1186/s12859-017-1947-7 (PMC5707825; doi:10.1186/s12859-017-1947-7)
Supplement: Additional file 1: Table S1. — Results for SCWRL + PROBE on validation dataset. (PDF 13 kb) [file 12859_2017_1947_MOESM1_ESM.pdf]

SupplementaryTable 1. Results for SCWRL+PROBE on validation dataset

Study	TP[b]	FP[b]	TN[b]	FN[b]	Total	NPV	PPV	Sensitivity	Specificity	Accuracy	MCC
CD40LG	7	0	19	6	32	0.68	1	0.53	1	0.76	0.60
SH2	11	27	44	17	99	0.51	0.51	0.40	0.62	0.51	0.02
ELANE	16	1	2	4	23	0.77	0.70	0.80	0.65	0.73	0.46
TP53	16	17	104	27	164	0.58	0.73	0.37	0.86	0.62	0.27
CANCER	3	3	18	7	31	0.55	0.67	0.29	0.86	0.57	0.18
Total/Average	53	48	187	61	349	0.60	0.70	0.48	0.80	0.64	0.29
